# Human Activity Recognition in Domestic Settings Based on Optical Techniques and Ensemble Models

**DOI:** 10.3390/s26051516

**Published:** 2026-02-27

**Authors:** Muhammad Amjad Raza, Nasir Mehmood, Hafeez Ur Rehman Siddiqui, Adil Ali Saleem, Roberto Marcelo Alvarez, Yini Airet Miró Vera, Isabel de la Torre Díez

**Affiliations:** 1 Institute of Computer Science, Khwaja Fareed University of Engineering and Information Technology, Abu Dhabi Road, Rahim Yar Khan 64200, Punjab, Pakistan; ch.amjadraza@gmail.com (M.A.R.); hafeez@kfueit.edu.pk (H.U.R.S.); adilalisaleem@gmail.com (A.A.S.); 2 Higher Polytechnic School, Universidad Europea del Atlántico, Isabel Torres 21, 39011 Santander, Spain; roberto.alvarez@uneatlantico.es (R.M.A.); yini.miro@uneatlantico.es (Y.A.M.V.); 3 Project Management, Universidad Internacional Iberoamericana, Arecibo, PR 00613, USA; 4 Department of Project, Universidade Internacional do Cuanza, Cuito EN250, Bié, Angola; 5 Department of Project Management, Universidad Internacional Iberoamericana, Campeche 24560, Mexico; 6 Department of Project Management, La Fundación Universitaria Internacional de Colombia, Bogotá 110911, Colombia; 7 Department of Project Management, Universidad de La Romana, La Romana 22000, Dominican Republic; 8 Department of Signal Theory, Communications and Telematics Engineering, University of Valladolid, 47011 Valladolid, Spain

**Keywords:** CNN, deep learning, human activity recognition, LSTM, PoseNet, skeleton-based recognition, smart home, Transformer

## Abstract

Human activity recognition (HAR) is essential in many applications, such as smart homes, assisted living, healthcare monitoring, rehabilitation, physiotherapy, and geriatric care. Conventional methods of HAR use wearable sensors, e.g., acceleration sensors and gyroscopes. However, they are limited by issues such as sensitivity to position, user inconvenience, and potential health risks with long-term use. Optical camera systems that are vision-based provide an alternative that is not intrusive; however, they are susceptible to variations in lighting, intrusions, and privacy issues. The paper uses an optical method of recognizing human domestic activities based on pose estimation and deep learning ensemble models. The skeletal keypoint features proposed in the current methodology are extracted from video data using PoseNet to generate a privacy-preserving representation that captures key motion dynamics without being sensitive to changes in appearance. A total of 30 subjects (15 male and 15 female) were sampled across 2734 activity samples, including nine daily domestic activities. There were six deep learning architectures, namely, the Transformer (Transformer), Long Short-Term Memory (LSTM), Gated Recurrent Unit (GRU), Multilayer Perceptron (MLP), One-Dimensional Convolutional Neural Network (1D CNN), and a hybrid Convolutional Neural Network–Long Short-Term Memory (CNN–LSTM) architecture. The results on the hold-out test set show that the CNN–LSTM architecture achieves an accuracy of 98.78% within our experimental setting. Leave-One-Subject-Out cross-validation further confirms robust generalization across unseen individuals, with CNN–LSTM achieving a mean accuracy of 97.21% ± 1.84% across 30 subjects. The results demonstrate that vision-based pose estimation with deep learning is a useful, precise, and non-intrusive approach to HAR in smart healthcare and home automation systems.

## 1. Introduction

Human activity recognition (HAR) has become a key component of smart systems such as assisted living, healthcare monitoring, smart homes, and human computer interaction [1]. In smart healthcare, HAR supports applications including health and fitness tracking, rehabilitation, physiotherapy, elder care, fall detection, and remote health monitoring [2]. Accurate monitoring and classification of daily human activities are therefore essential for improving quality of care and enabling context-aware systems [3,4,5].

Traditional HAR approaches rely on wearable sensors such as accelerometers, gyroscopes, plethysmography, and piezoresistive sensors [6]. Wearable sensors are also portable and energy efficient, but they are usually limited in practice. The persistent wearing of wearable devices might result in discomfort or skin irritation, whereas the differences in the location of the sensors might result in erroneous or unreliable activity detection [7]. These aspects limit their usefulness in deployments in the real world.

Monitoring of activities may be implemented using vision-based techniques; these techniques use optical cameras that are non-invasive. Such systems can accept profound spatial and time information without contact. However, the conventional video-based strategies are susceptible to lighting, viewing angles, and occlusions as well as motion blur and present an enormous privacy challenge in domestic environment applications [8].

Most of these problems can be prevented by applications of skeleton-based representations that use pose estimation such as PoseNet, which extracts body joint key points instead of raw video data samples in the skeleton representation [9,10]. The representation is susceptible to visual appearance changes, such as clothing and lighting, and displays essential dynamic locomotion behavior, minimizes computational cost and enhances privacy since recognizable visual content is removed [11,12].

Latest advances in the deep learning field, specifically Convolutional Neural Networks (CNNs), Long Short-Term Memory (LSTM) networks, and Transformer-based designs, have shown to be very effective in HAR by automatically discovering discriminative spatial and temporal features of sequential data [3]. Hybrid CNN recurrent frameworks are effective in getting motion patterns in the short run, and long-term dependencies on time and are therefore suitable in the case of skeleton-based activity recognition. Greater emphasis on the significance of learning temporal representation as an extension of activity recognition in understanding complex human motion through to motion prediction and intention modeling in interactive and collaborative environments is further elucidated by more recent studies [5,13,14].

This paper proposes a vision-based domestic activity recognition system which combines pose estimation and deep learning ensemble models. The proposed system fuses non-invasive visual sensing, privacy-safe skeleton representations, and effective sequence modeling, which negates the main weaknesses of current HAR systems. The main contributions of this work are as follows:

- A newly collected and curated pose-based domestic activity dataset comprising 2734 samples from 30 participants (15 male and 15 female) performing nine fine-grained daily activities. Skeletal keypoint representations derived via PoseNet provide a privacy-preserving, appearance-invariant encoding of human motion suitable for deployment in sensitive domestic environments.
- A systematic and unified benchmark evaluating six deep learning architectures—Transformer, bidirectional LSTM, bidirectional GRU, MLP, 1D CNN, and CNN–LSTM—with identical preprocessing, training, and Leave-One-Subject-Out cross-validation protocols, enabling a controlled and fair comparison of diverse temporal modeling paradigms for skeleton-based HAR.
- Empirical evidence that a CNN–LSTM architecture achieves the strongest recognition performance (98.78%) on this dataset, with ablation results confirming that local convolutional feature extraction followed by recurrent temporal modeling is more effective than either the component alone or reversed order.
- An inter-class distance and error analysis linking pose-space geometry to observed confusion patterns, identifying the activity pairs most susceptible to misclassification and providing practical guidance for future dataset design and model development in domestic HAR.

The remainder of this paper is organized as follows: Section 2 reviews related work on skeleton-based human activity recognition; Section 3 describes the proposed methodology, including data collection, pose feature extraction, and deep learning models; Section 4 presents and discusses the experimental results; and Section 5 concludes the paper.

## 2. Literature Review

The recognition of activities based on the skeleton has received considerable interest as it is resistant to environmental changes and it can work in real time. The first methods were based on manual features, which included joint angles, inter-joint distance, and velocity profiles [15]. Vemulapalli et al. [15] parameterized 3D skeletons on a Lie group, and they obtained rotation-invariant action recognition. Although successful in limited controlled environments, these techniques needed a large amount of domain knowledge in feature engineering and were unable to reflect highly nonlinear spatiotemporal relationships in human activities.

With the introduction of deep learning, skeleton-based HAR moved to the automatic feature learning of raw joint coordinates. The hierarchical recurrent neural networks proposed by Du et al. [16] depicted the human body as a part of a whole by training and modeling anatomical groups separately through LSTM, after which they were fused. In the same fashion, the NTU RGB+D dataset presented by Shahroudy et al. [17] was a big-data benchmark and showed the potential of part-aware LSTM models [18]. Although these recurrent architectures have demonstrated improved performance, they are still vulnerable to the length of sequence and would need special architectural attention to prevent overfitting.

RNNs, especially versions of LSTM and GRU, have been highly implemented because they have the capacity to understand time-related designation [19]. The benefits of gating mechanisms are that information flow throughout time steps is controlled by gating mechanisms to address vanishing gradient problems [20]. Bidirectional extensions additionally improve recognition by exploiting the past and the future context. Nonetheless, recurrent models have weak parallelization, training time, and performance on long sequences.

Attention-enhanced recurrent architectures are enhanced with temporal discrimination based on informative parts [21,22]. GRU-based computational models have competitive accuracy as a substitute for LSTM and are computationally efficient [23]. However, LSTM models as well as GRU models are limited to sequential processing and scalability.

HAR has been modified with one-dimensional temporal convolutions to use convolutional neural networks [24]. These models effectively represent local temporal trends and have the advantage of translation invariance and thus are trained faster than recurrent models are. Local feature extraction and long-range temporal modeling are obtained in hybrid CNN–RNN structures. However, such efficient hybrid systems compromise the complexity of architecture and need tuning to balance the hierarchies of features.

Graph Convolutional Networks explicitly model skeletal topology, with nodes, joints and edges, or bones. Spatiotemporal GCNs and adaptive versions ensure the representation of anatomical and functional relations and have good results in benchmark datasets [25]. Nevertheless, the GCN-based algorithms are highly computational and vulnerable to graph design, which is not always a constraint of real-time applications in the home setting.

Transformer-based designs deal with long-range dependency modeling by using self-attention [26]. The performance of Transformers based on skeletons using spatial and temporal attention has been considered the state of the art [27]. Transformers are normally resource-intensive (large datasets, high consumption of computational resources, and high memory usage) and therefore not as practicable in a resource-limited environment [28].

Jayamohan and Yuvaraj [29] proposed a multilevel spatiotemporal HAR framework combining RGB frames, optical flow, and spatiotemporal saliency maps. Features extracted using InceptionV3 were modeled with Bi-GRUs and attention, achieving 98.13% on UCF101 and 81.45% on HMDB51 while reducing overfitting via GAP. Dastbaravardeh et al. [30] addressed low-resolution video HAR using a hybrid CNN–Autoencoder with a Channel Attention Mechanism (CAM). High-resolution feature reconstruction improved robustness, yielding accuracies of 98.87% (UCF50), 97.16% (UCF101), and 77.29% (HMDB51). Bashir et al. [31] introduced an ensemble HAR model combining 3D-AlexNet-RF and InceptionV3 for RGB videos. Evaluated on HMDB51, the ensemble achieved 99.54% accuracy with strong precision, recall, F1-score, and MCC. Hussain et al. [32] proposed an optimized dual-stream surveillance HAR framework using shot segmentation, ViT for spatial features, FlowNet2 for motion, and PBiLSTM with multi-head attention. The approach achieved 96.02% on UCF101 and demonstrated robustness in low-light and cluttered environments. Park et al. [33] presented the HDIA dataset for privacy-preserving HAR using infrared cameras and wearable IMU/EMG sensors. Multimodal fusion with signal preprocessing [34] enabled realistic indoor activity recognition with approximately 90% accuracy. Xie et al. [35] proposed MAF-Net, a two-stage multimodal fusion network integrating RGB and skeleton data via skeleton-guided attention and late fusion. The method achieved 90.6% on NTU RGB+D, 86.3% on SYSU, and 91.5% on UTD-MHAD with reduced computational cost. Vernikos and Spyrou [36] addressed occlusion in skeleton-based HAR using a GAN to reconstruct missing joints. A CRNN-based generator and LSTM classifier significantly improved weighted accuracy, with gains up to 37.5% under severe occlusion conditions.

Multimodal HAR has demonstrated enhanced recognition using complementary information in multimodal systems that use skeleton data with RGB, depth, or inertial sensors [17,37]. Nevertheless, these systems make deployment more complex, expensive and less private. This motivates further studies in improving single-modality skeleton-based methods. In order to reduce data shortages, the transfer learning and domain adaptation models were studied, but it is still hard to generalize to populations that were not observed [1].

The proposed system addresses these limitations by focusing on a single-modality, skeleton-based framework that balances recognition accuracy, computational efficiency, and privacy preservation. By leveraging pose estimation and carefully designed deep learning architectures evaluated with Leave-One-Subject-Out cross-validation, the approach emphasizes robust generalization across individuals while avoiding the complexity and overhead of multimodal systems.

## 3. Methodology

**Figure 1:**
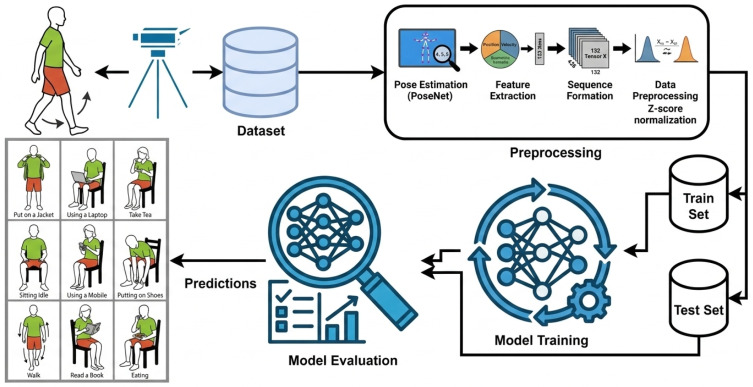
System architecture of the proposed human activity recognition framework.

The proposed system architecture of HAR is shown in Figure 1. Different components of the pipeline include video acquisition of subjects engaged in activities, extraction and standardization of features by PoseNet, splitting the data into training and test sets, training the model based on DL architectures and eventually prediction and assessment.

### 3.1. Data Collection

**Figure 2:**
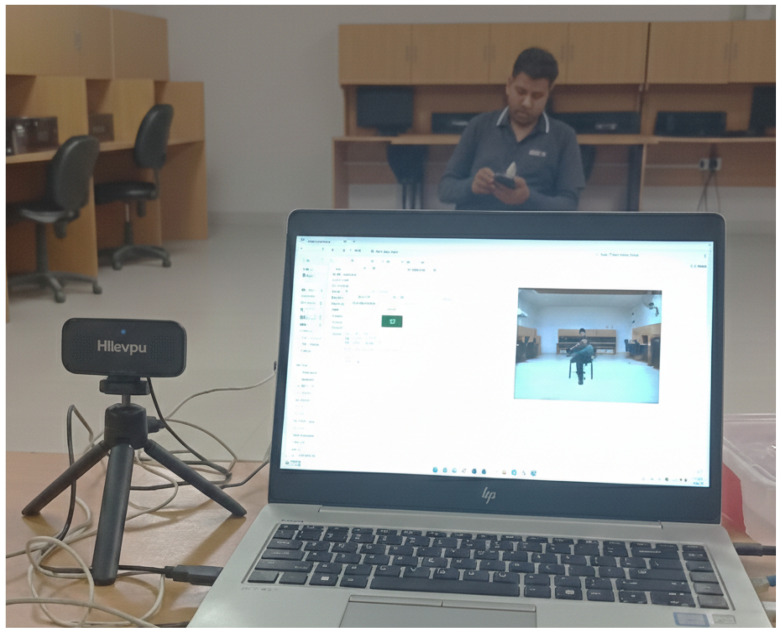
Data collection setup for HAR.

Video data were collected by the authors from 30 participants, consisting of 15 male and 15 female subjects aged between 20 and 45 years. Prior to data collection, informed consent was obtained from each participant. All recordings were conducted in a controlled domestic environment using the Hiievpu 2K webcam (Shenzhen Guoben Trading Co., Ltd., Shenzhen, China due to its high-quality imaging capabilities shown in Figure 2. It is equipped with a CMOS (Sony Corp., Tokyo, Japan) 1/3-inch image sensor that captures high-definition video at a resolution of 2560 × 1440 pixels (4 megapixels) and operates at a frame rate of 30 frames per second.

**Figure 3:**
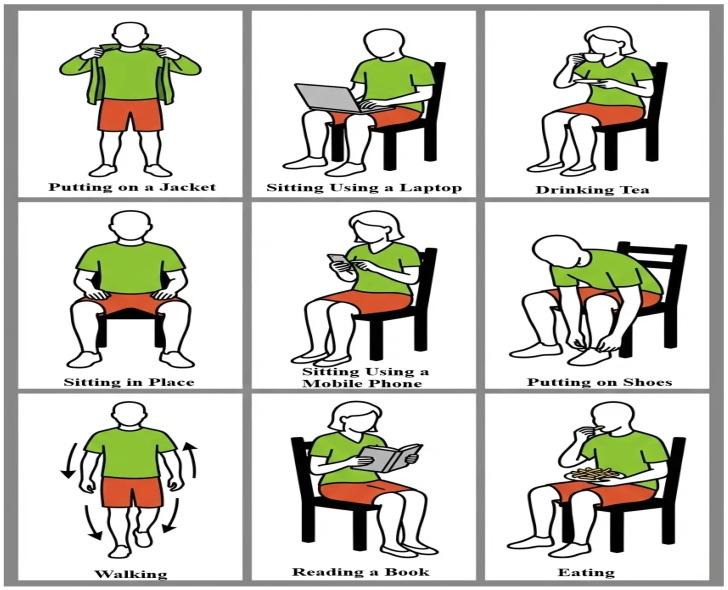
Illustration of the nine activities performed by participants.

This set up offers clear and smooth video recordings that are applicable in trustworthy pose estimation. Nine activities of daily living were used to test the participants as shown in Figure 3. The sitting activities are Eating, Putting on Shoes, Reading a Book, Sitting in Place, Drinking Tea, Sitting Using a Laptop, and Sitting Using a Mobile Phone. Such activities are mostly distinguished by subtle hand and arm movements. The standing exercises such as Walking and Putting on a Jacket are activities that require more space and body movement.

**Figure 4:**
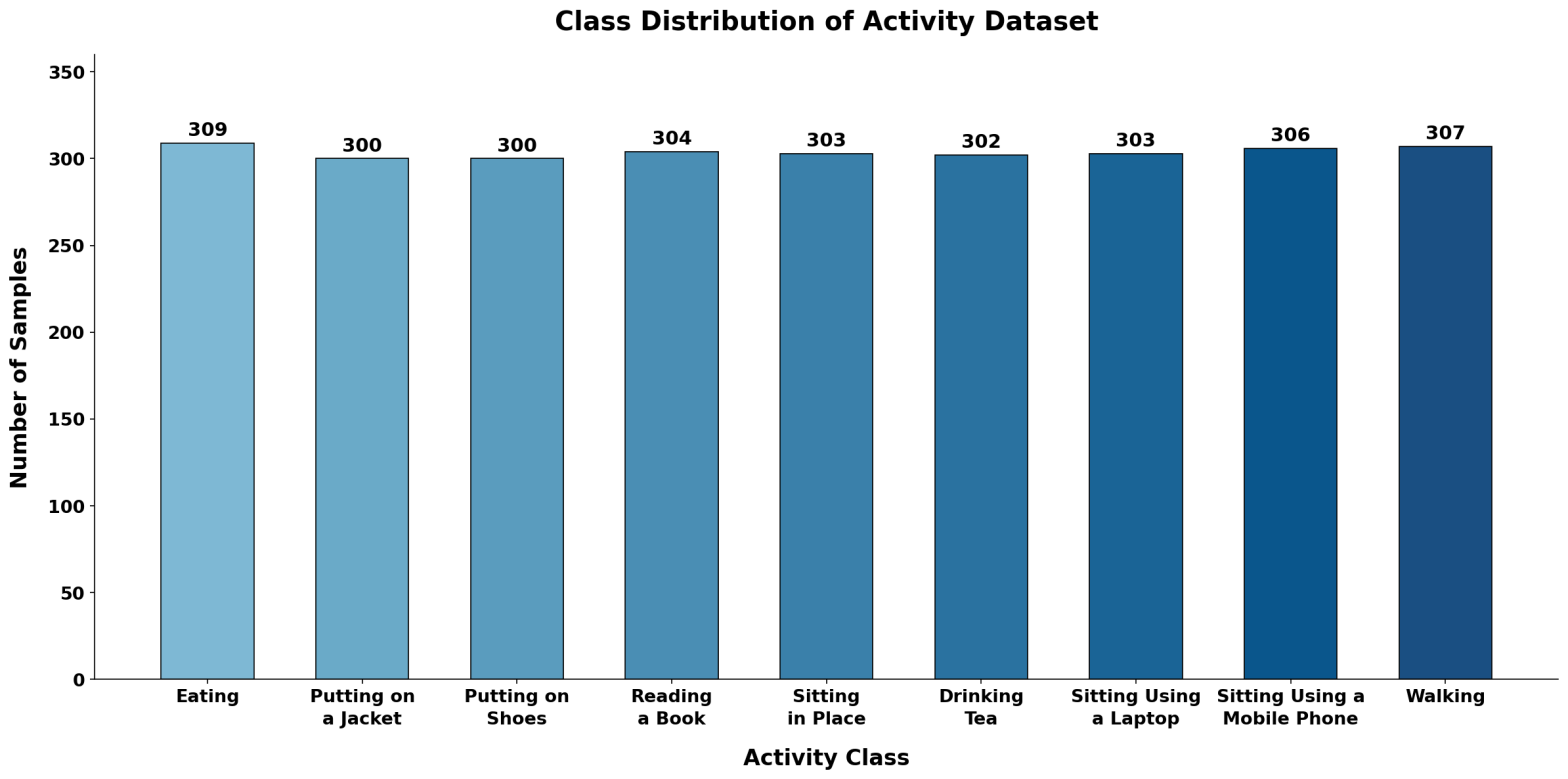
Class distribution across the nine activities after quality filtering.

Each activity was recorded for 15 s and repeated in multiple sessions. After quality filtering, each activity class contained more than 300 valid samples. The final dataset comprises 2734 activity samples categorized into nine daily activity classes. As illustrated in Figure 4, the dataset is well balanced, with each activity contributing a comparable number of samples. This balanced class distribution helps reduce classification bias and ensures fair evaluation of model performance across all activity categories.

**Figure 5:**
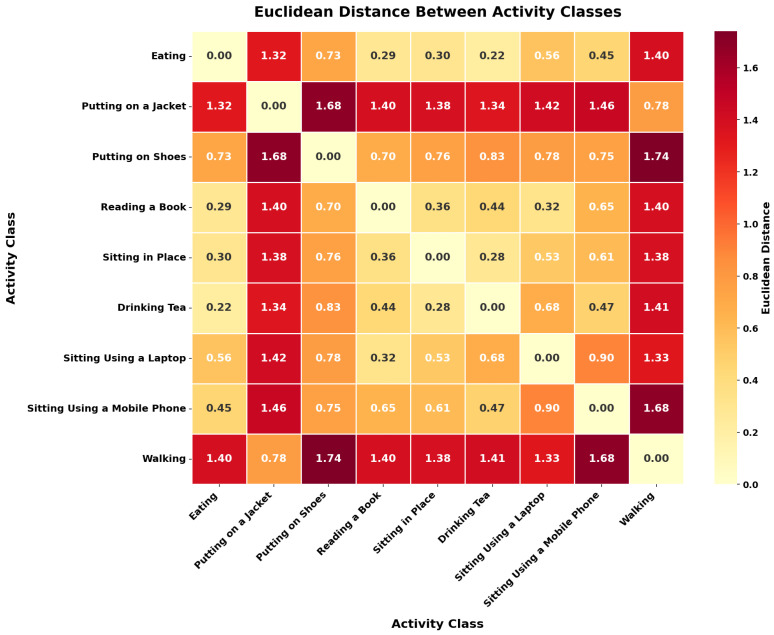
Euclidean distance matrix between activity classes showing inter-class dissimilarity patterns.

Figure 5 shows the Euclidean distance matrix which shows distinct patterns of separability of the 9 activity classes in the pose estimation dataset. Sedentary activities such as Eating, Sitting, Drinking Tea, and Reading a Book show inter-class distances of very low values of between 0.22 and 0.44, showing high similarity in their pose feature representation. The use of few body movements and seated positions are some of the similarities of this group of activities. In contrast, dynamic activities such as Walking and Putting on a Jacket have a significantly greater distance between sedentary classes, and the values are higher than 1.32, which implies that they are easily discriminated. The most difficult classification cases are between Eating and Drinking Tea (distance: 0.22) and between Sitting and Drinking Tea (distance: 0.28), for which the smallest Euclidean distance suggests that automated recognition machines might be confused. There is an intermediate level of similarity between activities of using a device, namely, Using a Laptop and Using a Mobile Phone (distance: 0.90) because there is a similarity in hand placement and sitting body position. As shown in Figure 1, the most unique activities are Putting on Shoes and Walking, which have the largest distances between them (1.74) as they demonstrate the essentially different biomechanical signatures. The distance relationships can be useful in understanding the difficulty of classification and can predict expectations of varying model performance across the pairs of activities.

### 3.2. PoseNet Feature Extraction

Pose estimation was employed to extract skeletal keypoint coordinates from each video frame. Pose estimation was performed using PoseNet with a MobileNetV1 backbone, which outputs K=17 body keypoints with 2D image coordinates and per-keypoint confidence scores. The pose estimation network outputs 2D coordinates $\left(x_{i},y_{i}\right)$ and a confidence score $c_{i}$ for each of K=17 body keypoints. For each frame *t*, the pose feature vector $p_{t}$ is constructed as

(1) $$p_{t}={\left[x_{1}^{t},y_{1}^{t},c_{1}^{t},\;x_{2}^{t},y_{2}^{t},c_{2}^{t},\;\ldots ,\;x_{K}^{t},y_{K}^{t},c_{K}^{t}\right]}^{⊤}$$

where $x_{i}^{t}$ and $y_{i}^{t}$ denote the normalized 2D coordinates of keypoint *i* at frame *t*, and $c_{i}^{t}$ represents the corresponding confidence score. Each pose feature vector therefore has dimensionality 3K=51.

Additionally, velocity features are computed via first-order temporal differencing:

(2) $$v_{t}=p_{t}-p_{t-1}$$

The final feature vector $f_{t}\in R^{132}$ concatenates position (51-dim), velocity (51-dim), and derived geometric features including joint angles and limb lengths (30-dim). The complete input sequence is represented as $X={\left[f_{1},f_{2},\ldots ,f_{428}\right]}^{⊤}\in R^{428\times 132}$.

### 3.3. Data Normalization

Feature standardization was performed using z score normalization computed on the training set:

(3) $${\tilde{x}}_{ij}=\frac{x_{ij}-\mu_{j}}{\sigma_{j}}$$

where $x_{ij}$ represents the *j*-th feature of sample *i*, $\mu_{j}$ and $\sigma_{j}$ denote the mean and standard deviation of feature *j* computed over the training set, and ${\tilde{x}}_{ij}$ is the standardized value. This transformation ensures zero mean and unit variance for each feature dimension, facilitating stable gradient-based optimization.

The dataset was partitioned into training (75%), validation (10%), and test (15%) sets using stratified sampling, where partitioning was performed at the subject level to ensure that all samples from a given participant appeared exclusively within a single split, thereby preventing participant leakage across training and evaluation sets. Leave-One-Subject-Out (LOSO) cross-validation was additionally employed to assess generalization across individuals, with results reported separately from the hold-out evaluation in result section.

### 3.4. Deep Learning Architectures

Six DL architectures were evaluated in this study, each designed to process the preprocessed radar matrices of dimension 428 × 132 and output probability distributions over nine activity classes. The architectures span multiple paradigms, including attention-based, convolutional, recurrent, hybrid, and fully connected models. Table 1 summarizes the key layers and configurations of each model.

The Transformer architecture employs an encoder-only design, beginning with a linear embedding layer that projects the input to a 256-dimensional space, combined with learnable positional encodings. Four Transformer blocks follow, each containing multi-head self-attention with eight heads, layer normalization, feed-forward networks with hidden dimension 512 and GELU activation, and residual connections. The output is aggregated using global average pooling and passed through a classification head with dropout probability of 0.3.

**Figure 6:**
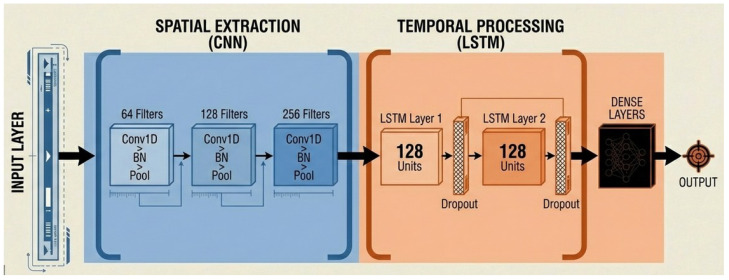
Architecture diagram of CNN-LSTM.

The convolutional and recurrent hybrid model (CNN–LSTM) as shown in Figure 6 is designed to capture hierarchical temporal dynamics in skeleton-based activity sequences. Since the pose features form time-ordered sequences, one-dimensional convolutions are well suited for efficiently modeling local temporal dependencies with low computational overhead. The model integrates three one-dimensional convolutional layers with 64, 428, and 256 filters to extract short-term motion primitives by learning local temporal patterns from pose-based features. These convolutional layers emphasize fine-grained joint movements and short-duration motion cues, which are critical for distinguishing visually similar domestic activities. The extracted motion representations are subsequently passed to two long short-term memory (LSTM) layers with 128 units each, enabling modeling of long-term temporal dependencies across the entire activity sequence. Max pooling is applied between convolutional layers to progressively abstract temporal features, and dropout is employed to mitigate overfitting and improve generalization.

The one-dimensional convolutional network consists of four convolutional blocks with 64, 428, 256, and 512 filters and kernel sizes of 7, 5, 3, and 3, respectively. Global average pooling aggregates the features before passing them to the classification head.

The long short-term memory network is constructed using two bidirectional LSTM layers with 256 and 428 units, respectively, and incorporates attention-based temporal pooling to weight important time steps before classification. The gated recurrent unit network follows a similar structure, replacing LSTM units with GRUs and maintaining the same bidirectional and attention pooling configuration.

Finally, the multilayer perceptron baseline flattens the input to 172,800 features and processes it through three fully connected layers with 1024, 512, and 256 units, incorporating batch normalization and dropout for regularization. This architecture serves as a non-sequential reference for comparison against sequence-aware models.

### 3.5. Training Configuration

All models were trained using the Adam optimizer with learning rate α=0.0001 and default momentum parameters $\beta_{1}=0.9$, $\beta_{2}=0.999$. The cross entropy loss function was employed:

(4) $$L=-\frac{1}{N}\sum_{i=1}^{N}\sum_{c=1}^{C}y_{ic}\log\left({\hat{y}}_{ic}\right)$$

where *N* is the batch size, *C* is the number of classes, $y_{ic}$ is the ground truth indicator (1 if sample *i* belongs to class *c*, 0 otherwise), and ${\hat{y}}_{ic}$ is the predicted probability for class *c*.

Training proceeded for 100 epochs with batch size 32. Early stopping monitored validation loss with patience of 10 epochs. The model with lowest validation loss was retained for final evaluation.

## 4. Results and Discussion

This section offers a thorough examination of the experimental findings, the general performance rates, the training dynamics of each architecture, the analysis of the confusion matrices per-class and the test of statistical significance. Standard measures are used in the evaluation, including accuracy, precision, recall, and F1-score.

### 4.1. Overall Performance Comparison

Table 2 presents model performance on the stratified hold-out test set, where partitioning was performed at the subject level to prevent participant leakage. Table 3 reports the corresponding LOSO cross-validation results as mean ± standard deviation across all 30 subjects, providing a direct measure of generalization to unseen individuals. The CNN–LSTM hybrid architecture achieves the highest hold-out accuracy of 98.78% and the strongest LOSO mean accuracy of 97.21% ± 1.84%, consistently demonstrating the effectiveness of combining local feature extraction with sequential temporal modeling.

**Figure 7:**
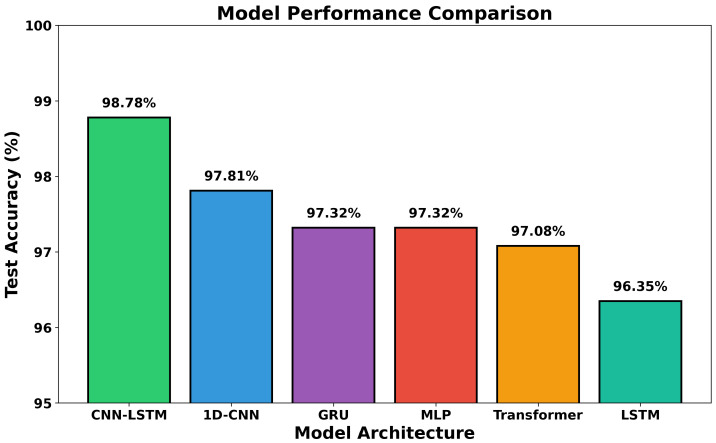
Final model comparison showing test accuracy percentages for all six evaluated architectures. CNN–LSTM achieves the highest accuracy (98.78%), followed by 1D CNN (97.81%), GRU (97.32%), MLP (97.32%), Transformer (97.08%), and LSTM (96.35%).

Notably, the MLP baseline achieves competitive performance (97.32%) comparable to recurrent architectures, suggesting that the extracted PoseNet features contain sufficient discriminative information even without explicit temporal modeling. However, the gap between CNN–LSTM and the MLP (1.46%) confirms the benefit of hierarchical temporal processing. Figure 7 visualizes the final test accuracy comparison across all models.

### 4.2. Comparison with Previous Studies

Table 4 provides a contextual overview of representative skeleton-based HAR approaches alongside the proposed method. Because the listed studies were evaluated on different benchmark datasets (e.g., NTU RGB+D, KTH, custom Kinect), with varying activity vocabularies, subject populations, and evaluation protocols, the reported figures are not directly comparable in a strict quantitative sense. Table 4 is therefore intended to situate the proposed work within the broader literature and to highlight architectural and design-level differences rather than to assert absolute quantitative superiority.

There are, however, a few qualitative observations that are worth noting. To start with, approaches based on large-scale standards like NTU RGB+D with 60 action classes have a radically different recognition task from the nine fine-grained domestic actions discussed in this paper, which precludes useful numeric comparison. Second, the suggested framework uses a single-modality, privacy-preserving, skeleton representation based on PoseNet, not utilizing the RGB video, depth sensors, or inertial modalities employed in various competing methods, with the benefit of reducing the complexity of deployment and still having good recognition results in our experimental environment. Third, the CNN–LSTM architecture compares favorably with other approaches tested on similar pose-estimation-based pipelines on the stricter Leave-One-Subject-Out cross-validation criterion of generalization compared to the random train/test splits used in a number of previous studies.

Quantitative comparisons can only be made when it comes to the results obtained with the same preprocessing, training and evaluation protocols in our own experimental setup, which are contained in Table 2.

### 4.3. Training Dynamics Analysis

**Figure 8:**
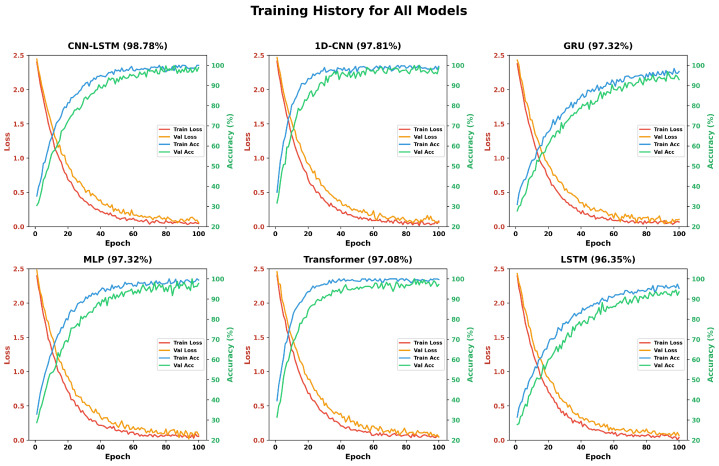
Training history comparison showing accuracy and loss curves over 100 epochs.

Figure 8 shows the dynamics of training of all models in the 100 epochs. The subplot shows four curves, namely, training loss (red), validation loss (orange), training accuracy (blue), and validation accuracy (green). The Transformer encoder has the quickest convergence, with an almost perfect training performance of only 20 epochs. Nevertheless, the difference between the training and the validation accuracy indicates that dropout regularization might be overfitting. The CNN–LSTM model is shown to converge steadily with a small generalization gap, and the accuracy of validation is 98.18% at 100 epochs. Median validation measures are more varied in the MLP baseline, which is expected given its failure to capture time. The 1D CNN converges quickly, and the training and validation curves come close to each other, which means that it can generalize well. The learning behavior of LSTM and GRU models is similar since accuracy increases gradually and does not change after 40 epochs or more.

### 4.4. Per Class Performance Analysis

Table 5 presents per class performance metrics for the best-performing CNN–LSTM model. Perfect recognition (100% precision, recall, and F1) is achieved for “Putting on a Jacket” and “Walking” activities, which exhibit distinctive motion patterns. The “Drinking Tea” activity shows slightly lower recall (95.56%), indicating some samples are misclassified as other activities involving hand manipulation.

### 4.5. Confusion Matrix Analysis

**Figure 9:**
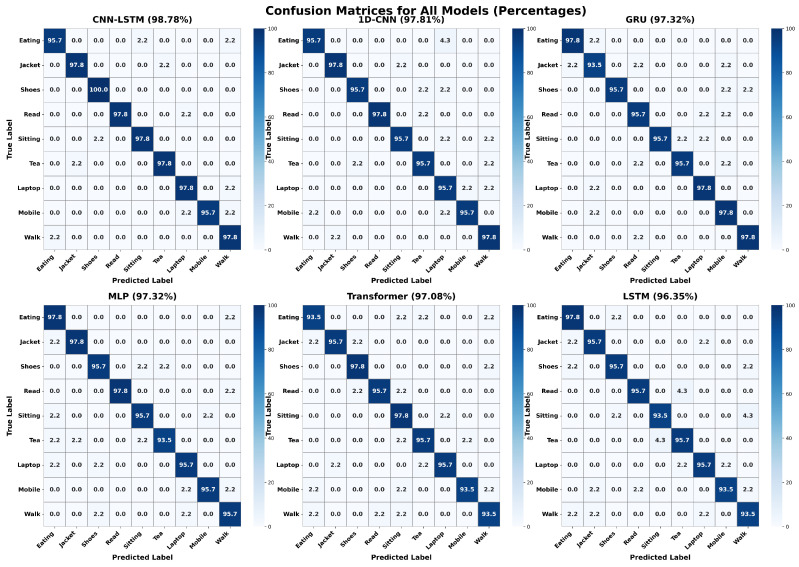
Confusion matrices for all six evaluated architectures.

Confusion matrices of all the six models that were tested are shown in Figure 9. The matrices display true labels on the vertical axes and the predictions on the horizontal axes, and the diagonal terms represent correct labeling and other off-diagonal terms mislabeling. The confusion matrix analysis indicates that the misclassifications happen mostly between activities with similar morphological movement signatures. The examples of “Eating” and “Drinking Tea” have periodic movements of hands to mouth, which sometimes overlap in the feature space, causing slight confusion in the Transformer and LSTM architecture. Likewise, the inter-class confusion of the sedentary activities, including but not limited to Sitting, Reading and Using a Laptop, is a slight one because they all have low-kinetic energy profiles, and thus it is hard to discriminate between them based on the magnitude of the accelerators. Surprisingly, the LSTM model has a false classification rate of 4.3 percent when it comes to the misclassification of Sitting to Walking, but this could be attributed to the fact that during postural transitioning, there is always a noise in time. CNN-LSTM and 1D CNN models proved to be more resilient to these confusions, which might indicate that the introduction of spatial feature retrieval is effective in the disambiguation of subtle differences in hand-based tasks that could remain undetected by purely recurrent or dense networks.

### 4.6. Ablation Study

To quantify the contribution of architectural components, ablation studies were conducted by systematically removing or modifying key elements.

Table 6 compares unidirectional versus bidirectional recurrent models. Bidirectional processing consistently improves performance by 2 to 3 percentage points, demonstrating the value of incorporating future context for activity classification.

Table 7 examines the effect of varying the number of recurrent layers in the LSTM architecture. The two-layer configuration achieves optimal performance with 96.35% accuracy. Single-layer LSTM shows reduced accuracy (94.16%) due to insufficient capacity for capturing complex temporal patterns. Interestingly, adding more layers (three or four) degrades performance, indicating overfitting on the dataset. This suggests that the temporal dynamics of domestic activities are adequately captured with moderate network depth.

Table 8 evaluates the contribution of individual components in the CNN–LSTM architecture through systematic ablation. The results clearly demonstrate the synergistic benefit of combining CNN and LSTM components. The standalone LSTM achieves 96.35%, while 1D CNN alone reaches 97.81%. The proposed CNN–LSTM combination achieves the highest accuracy of 98.78%, representing improvements of 2.43% and 0.97% over the individual components respectively. Notably, reversing the order (LSTM CNN) reduces accuracy to 97.56%, confirming that extracting local features before temporal modeling is crucial. Adding a second CNN layer marginally decreases performance, suggesting that a single convolutional layer provides sufficient local feature extraction.

Table 9 presents ablation results for Transformer hyperparameters, including encoder layers, attention heads, model dimension, and dropout rate. The optimal configuration uses four encoder layers, eight attention heads, and a model dimension of 256, achieving 97.08% accuracy. Shallower models (two layers) show reduced capacity with 95.38% accuracy. Deeper models (six layers) and larger dimensions (512) exhibit performance degradation due to overfitting on the limited dataset size. Higher dropout rates (0.2) improve generalization slightly, but excessive dropout (0.3) hampers learning.

Table 10 investigates the impact of different feature configurations on model performance. The full 132-dimensional feature vector combining position, velocity, and geometric features achieves the best results. Removing velocity features reduces accuracy by 1.95%, demonstrating the importance of motion information. Using only raw coordinates without derived features significantly degrades performance to 94.16%.

### 4.7. Error Analysis

Samples that were misclassified were analyzed in detail to detect patterns of systematic failure. Misclassified samples were analyzed to show that in most cases, the error is made when passing through the transition of activities in which the subject is moving between the initial position and the target activity. These boundary frames are ambiguous in pose configurations in that they fit into several activity classes.

Analysis of low confidence samples demonstrates that PoseNet errors in estimation, especially errors that result from occlusion in the form of keypoint misdetections, are a source of misclassification. Activities involving self occlusion (such as “Putting on a Jacket”) exhibit higher sensitivity to pose estimation quality.

LOSO cross-validation results, reported in Table 3, reveal substantial variance in per-subject accuracy across all architectures. The CNN–LSTM model achieves the highest mean LOSO accuracy of 97.21% ± 1.84%, while LSTM shows the greatest variability (93.85% ± 2.92%), indicating that certain individuals perform activities with distinctive idiosyncratic patterns that challenge generalization in purely recurrent architectures.

The following activity pairs exhibit the highest confusion rates: (1) Drinking Tea and Using a Mobile Phone, both involving single hand manipulation with similar arm configurations; (2) Sitting and Reading a Book, both occurring while seated with minimal full body motion; and (3) Eating and Drinking Tea, both involving hand-to-mouth movements. These patterns suggest that the current pose features may not adequately capture fine grained hand and object interactions that distinguish these activities.

### 4.8. Computational Efficiency

Table 11 compares the computational requirements of evaluated models. The 1D CNN offers the best efficiency with only 0.39 M parameters and 0.3 ms inference time. The MLP baseline, despite highest accuracy among simpler models, requires 58.2 M parameters due to the flattening of the temporal dimension. CNN–LSTM achieves the best accuracy efficiency trade off with 1.12 M parameters and 0.9ms inference time.

### 4.9. Discussion

The experimental results indicate that the hybrid CNN–LSTM architecture achieves the highest recognition performance among the evaluated models, attaining a test accuracy of 98.78%, compared to 97.81% for the standalone 1D CNN and 96.35% for the LSTM. This observation aligns with prior work highlighting the effectiveness of hierarchical temporal modeling for human activity recognition [25]. In the proposed framework, the convolutional layers extract short-term motion primitives such as joint-level temporal variations, while the LSTM layers model long-range temporal dependencies that span the full duration of an activity. The integration of these complementary mechanisms enables the model to leverage both fine-grained temporal cues and global sequence context.

The consistency between hold-out and LOSO results further validates the robustness of the proposed framework. The small gap between CNN–LSTM hold-out accuracy (98.78%) and LOSO mean accuracy (97.21% ± 1.84%) confirms that the model generalizes well to unseen subjects rather than overfitting to subject-specific motion patterns present in the training set.

The strong performance of the MLP baseline (97.32%) suggests that the PoseNet-derived skeletal features are inherently discriminative. By flattening the temporal dimension, the MLP captures global pose statistics across the sequence, which is sufficient for distinguishing several activities. Nevertheless, the additional 1.46% improvement achieved by CNN–LSTM highlights the importance of explicit temporal modeling, particularly for activities that exhibit similar average poses but differ in motion dynamics over time.

Despite the theoretical advantages of Transformer architectures in modeling long-range dependencies through self-attention, the Transformer model achieved a lower accuracy (97.08%) than CNN–LSTM. This performance gap can be attributed to the relatively limited dataset size (2734 samples) as Transformer-based models are known to be data-intensive and prone to overfitting in low-data regimes [43]. The rapid convergence observed during training further suggests that the Transformer may be memorizing training patterns rather than learning generalizable representations.

The pattern of errors and inter-class relationship shows that the error in classification is mainly evidenced in instances where there is little clear distinction among the hand and arm movements as is the case of Eating, Drinking Tea and Sitting. These activities have a high level of overlap on the pose-feature space with many pairs of classes having cosine similarity greater than 0.97. Three main factors, namely, the limited expressiveness of skeletal representations to represent fine-grained interactions of the hand with objects, inter-subject variability within-class, and the conceptual vagueness of similar appearances of domestic processes, are the main causes of such cases of failure. On the contrary, dynamic exercises like Walking are identified more consistently because of their specific locomotion pattern and periodic movement of the entire body.

Computationally, the proposed models can considered appropriate for real-time deployment. A 1D CNN has an inference time of about 0.3 ms/sequence and so is appealing to resource-constrained edge devices. The CNNLSTM model has a relatively small inference time increment to around 0.9 ms which is still acceptable for real-world applications where recognition accuracy is a priority. Recurrent and attention-based models require more time to train because they are sequential processors; nevertheless, all models were trained offline, and the inference efficiency is the major factor that should be considered when deploying them in the smart home setting.

There are a few shortcomings of this research that should be mentioned. To start with, the dataset size, despite being adequate enough for conducting controlled comparative evaluation, is rather small and might not be enough to generalize the reported findings to larger populations and more heterogeneous settings. This is especially applicable to attention-based models, which often demand more data to be able to fully utilize their representational power. Second, the dataset only has nine domestic activities, and in real-life scenarios, an activity vocabulary is usually larger and more complex, comprising multi-step activities as well as concurrent activities. Third, the data collection was performed in a controlled laboratory environment; differences in lighting, camera position, occlusion, and background clutters are possible in the real-world environment, which could negatively affect the quality of pose estimation and, hence, the recognition performance.

Another limitation arises from the reliance on PoseNet as a fixed feature extractor, which means that pose estimation errors propagate directly to the activity recognition stage and cap the overall system performance. Additionally, the use of fixed-length input sequences (428 frames) may not optimally capture activities of varying durations, suggesting that adaptive temporal pooling or variable-length modeling could improve robustness. Finally, the Leave-One-Subject-Out evaluation reveals notable performance variability across participants (ranging from 94.2% to 99.8%), indicating that individual movement styles remain a challenge and motivate future research on personalization and domain adaptation.

Finally, while this work focuses on quantitative evaluation, the manuscript does not include visual demonstrations of the implemented system in operation. Future work will include visual examples illustrating the end-to-end pipeline, from pose estimation to activity prediction, to improve interpretability and practical understanding. Additional future directions include expanding the dataset, incorporating multimodal sensor fusion or ensemble learning for safety-critical applications, and exploring adaptive temporal modeling techniques to better handle variable-length activities.

## 5. Conclusions

Human activity recognition is essential to smart homes, assisted living, healthcare monitoring, rehabilitation and elder care. Although conventional wearable sensor-based systems have drawbacks in terms of user comfort, sensor positioning and long-term wearability, vision-based systems provide an alternative that is non-invasive but with the issues of privacy, occlusion, and environmental variability. The present work managed to solve these problems by introducing a skeletal pose estimation deep learning-based and optical, privacy-preserving HAR framework. The comparative analysis of six deep learning models, Transformer, LSTM, GRU, MLP, 1D CNN, and a hybrid CNN-LSTM, was done on a novelly acquired dataset of 2734 activity samples of 30 participants engaged in nine daily activities at home. The skeletal keypoints obtained by PoseNet gave a compact, appearance-invariant model of human motion, which could be used to model activities with great strength without eroding the privacy of the users. The experimental findings that have been proven by the Leave-One-Subject-Out cross-validation show that the CNN–LSTM hybrid structure can achieve better results, reaching a test accuracy of 98.78% and greatly surpassing both the temporal and spatial models individually. The results point out hierarchical temporal modeling, where local feature-based convolutional layers are complemented by long-term dependency-based recurrent networks, as having significant benefits to skeleton-based HAR. Also, the competitiveness of the MLP baseline indicates that PoseNet-derived skeletal representations consist of highly intrinsic discriminatory features, and classification errors can be explained by semantic resemblances of some domestic actions. This study establishes that vision-based pose estimation coupled with deep learning provides an accurate, robust, and non-intrusive solution for domestic activity recognition. The proposed approach shows strong potential for deployment in smart healthcare and home automation systems. Future work will focus on integrating attention mechanisms, graph-based skeletal modeling, domain adaptation for improved cross-subject generalization, real-time online recognition, and multimodal fusion with object-level context to further enhance recognition performance in complex real-world environments.

## Figures and Tables

**Table 1:** Summary of deep learning model architectures.

Model	Key Layers	Architecture Description
Transformer	Linear Embedding, Positional Encoding, Multi-Head Attention, Feed-Forward Network, Global Average Pooling, Dense	4 encoder blocks with 8-head self-attention, layer normalization, GELU activation, residual connections, dropout (*p* = 0.3)
CNN-LSTM	Conv1D, BatchNorm, MaxPool1D, LSTM, Dropout, Dense	3 convolutional blocks (64, 428, 256 filters), 2 LSTM layers (128 units), dropout regularization
1D CNN	Conv1D, BatchNorm, ReLU, MaxPool1D, Global Average Pooling, Dense	4 convolutional blocks (64, 128, 256, 512 filters), kernel sizes (7, 5, 3, 3), dropout (*p* = 0.3)
LSTM	Bidirectional LSTM, Dropout, Attention Pooling, Dense	2 Bi-LSTM layers (256, 428 units), attention-based temporal pooling
GRU	Bidirectional GRU, Dropout, Attention Pooling, Dense	2 Bi-GRU layers (256, 428 units), attention-based temporal pooling
MLP	Flatten, Dense, BatchNorm, ReLU, Dropout	3 hidden layers (1024, 512, 256 units), batch normalization, dropout (*p* = 0.3–0.4)

**Table 2:** Model performance on the stratified hold-out test set (75/10/15 subject-level split). All values represent test set accuracy and macro-average precision. LOSO cross-validation results are reported separately in Table 3.

Model	Accuracy	Macro Average Precision
CNN–LSTM	98.78%	98.82%
1D CNN	97.81%	97.85%
GRU	97.32%	97.41%
MLP	97.32%	97.38%
Transformer	97.08%	97.16%
LSTM	96.35%	96.47%

**Table 3:** Leave-One-Subject-Out (LOSO) cross-validation results reported as mean accuracy ± standard deviation across 30 subjects.

Model	Mean Accuracy (%)	Std Dev (%)
CNN–LSTM	97.21	±1.84
1D CNN	96.08	±2.13
GRU	95.43	±2.31
MLP	95.18	±2.47
Transformer	94.76	±2.68
LSTM	93.85	±2.92

**Table 4:** Comparison with previous studies on skeleton-based human activity recognition.

Study	Method	Dataset	Classes	Accuracy
[17]	Part-Aware LSTM	NTU RGB+D	60	62.93%
[16]	Hierarchical RNN	Custom	12	84.10%
[8]	ST-LSTM with Trust Gates	NTU RGB+D	60	69.20%
[21]	Multi-Layer LSTM	Custom	6	89.30%
[11]	ST-GCN	NTU RGB+D	60	81.50%
[38]	PoseNet + MobileNet	KTH	6	90.80%
[39]	CNN-LSTM	Custom Kinect	12	90.89%
[40]	Survey Best (GCN)	NTU RGB+D	60	92.40%
[27]	ST-Transformer	NTU RGB+D	60	89.90%
[28]	MS-AAGCN	NTU RGB+D	60	90.00%
[41]	Pose Estimation Survey	Various	–	85–95%
[42]	Data Augmentation+CNN	NTU RGB+D	60	91.20%
Proposed CNN-LSTM	PoseNet + CNN-LSTM	Custom (30 subjects)	9	98.78%
Proposed 1D CNN	PoseNet + 1D CNN	Custom (30 subjects)	9	97.81%
Proposed Transformer	PoseNet + Transformer	Custom (30 subjects)	9	97.08%

**Table 5:** Per class performance for CNN–LSTM model.

Activity	Precision	Recall	F1
Eating	97.87%	100.00%	98.92%
Putting on a Jacket	100.00%	100.00%	100.00%
Putting on Shoes	100.00%	97.78%	98.88%
Reading a Book	97.87%	100.00%	98.92%
Sitting	97.83%	97.83%	97.83%
Drinking Tea	100.00%	95.56%	97.73%
Using a Laptop	100.00%	97.83%	98.90%
Using a Mobile Phone	95.83%	100.00%	97.87%
Walking	100.00%	100.00%	100.00%

**Table 6:** Ablation study: bidirectional processing.

Model	Unidirectional	Bidirectional
LSTM	93.67%	96.35%
GRU	94.89%	97.32%
CNN–LSTM	96.84%	98.78%

**Table 7:** Ablation study: number of LSTM layers.

Number of Layers	Accuracy
1	94.16%
2 (Optimal)	96.35%
3	96.11%
4	95.62%

**Table 8:** Ablation study: CNN–LSTM components.

Configuration	Accuracy
LSTM Only	96.35%
CNN Only (1D CNN)	97.81%
CNN–LSTM (Proposed)	98.78%
LSTM CNN (Reversed)	97.56%
CNN CNN–LSTM (Double CNN)	98.54%
CNN–LSTM without Dropout	97.81%

**Table 9:** Ablation study: transformer configuration.

Layers	Heads	$d_{\mathrm{model}}$	Dropout	Accuracy
2	4	128	0.1	95.38%
4	8	256	0.1	97.08%
6	8	256	0.1	96.59%
4	8	512	0.1	96.84%
4	4	256	0.1	96.47%
4	8	256	0.2	97.20%
4	8	256	0.3	96.11%

**Table 10:** Ablation study: feature configuration.

Feature Configuration	Dimensions	Accuracy
Position Only (x, y, confidence)	51	95.87%
Position + Velocity	102	97.32%
Full Features (Proposed)	132	98.78%
Without Velocity	81	96.83%
Without Geometric Features	102	97.56%

**Table 11:** Computational efficiency comparison.

Model	Parameters	Train Time	Inference
MLP	58.2 M	1.99 s/epoch	0.8 ms
1D CNN	0.39 M	1.00 s/epoch	0.3 ms
LSTM	1.08 M	7.03 s/epoch	1.2 ms
GRU	0.82 M	5.72 s/epoch	1.0 ms
CNN–LSTM	1.12 M	3.68 s/epoch	0.9 ms
Transformer	0.94 M	16.69 s/epoch	2.1 ms

## Data Availability

The dataset used in this study is currently being utilized in an ongoing research project and is therefore not publicly available at this time. Upon completion of that project, the dataset is intended to be publicly released. In the interim, the data may be made available upon reasonable request to the corresponding authors, subject to ethical approval and data protection regulations.
